# Hemicrania Continua With Scintillating Scotoma: A Rare Presentation

**DOI:** 10.7759/cureus.10680

**Published:** 2020-09-27

**Authors:** Adil Hussein, Tham Han Shu, Mei Fong Chong, Chun Fai Cheah

**Affiliations:** 1 Ophthalmology, School of Medical Sciences, Universiti Sains Malaysia, Kubang Kerian, MYS; 2 Ophthalmology, Hospital Raja Permaisuri Bainun, Ipoh, MYS; 3 Neurology, Hospital Raja Permaisuri Bainun, Ipoh, MYS

**Keywords:** hemicrania continua, trigeminal autonomic cephalalgia, headache with aura, scintillating scotoma

## Abstract

Headache can be a primary or secondary disorder. The characteristics of headache and its associated features, especially the presence of red flag signs, are important in distinguishing secondary from primary causes. Hemicrania continua is a type of primary headache disorder characterized by a continuous unilateral headache with episodes of exacerbations and association with cranial autonomic symptoms, which include several ocular symptoms. The absolute response to indomethacin remains the hallmark of this disease. We would like to report a rare case of hemicrania continua with scintillating scotoma during exacerbations apart from the typical autonomic features of conjunctival injection, ptosis, eyelid edema, and lacrimation.

## Introduction

Hemicrania continua (HC) is a rare primary headache disorder. It is grouped under trigeminal autonomic cephalalgia after the revision of diagnostic criteria in the International Classification of Headache Disorders, 3rd edition (ICHD-3) Beta in 2013 [1], which include the characteristics of persistent unilateral chronic headache of more than three months, with episodes of exacerbations that respond absolutely to indomethacin. The autonomic features associated include eyelid edema, conjunctival injection with lacrimation, miosis and/or ptosis, rhinorrhoea, and facial flushing. The diagnostic criteria had been revised in 2013 based on the study reported cases with wider autonomic features and a side-shifting headache [2]. Migrainous features had been presented as part of hemicrania continua, however, visual aura was not commonly reported. By far, there have been only single reports or case series of hemicrania continua with visual symptoms mainly described as flashes or black spots during pain exacerbations [2-3]. Hence, we report a rare case of HC associated with documented scintillating scotoma as visual aura apart from the presence of typical autonomic features.

## Case presentation

A 22-year-old Indian lady presented with acute exacerbation of left-sided headache over the frontoparietal region and left eye pain for one day. The pain was throbbing in nature. It was associated with mild drooping and swelling of the left upper eyelid, eye redness, tearing, photophobia, and reduced periphery vision over the left eye. The headache was partially relieved by vomiting; otherwise, there was no body weakness and no history of trauma. She denied any blurring of vision and diplopia. Further history revealed that she had been having persistent headaches with similar recurrent episodes of exacerbations of left-sided headache and left eye pain for the past one year with similar associated ocular symptoms and nasal congestion. The visual disturbances of reduced periphery vision and photophobia occurred during headache exacerbations. It began at the paracentral region, gradually enlarged towards the periphery. The visual symptoms lasted for about 24 hours together with the headache spikes and resolved when the headache was partially relieved by bouts of vomiting. There was no associated phonophobia and no identifiable aggravating factor for the headache. She denied a history of overuse of analgesics. She had no personal and family history of migraine.

Upon initial presentation, visual acuity over both eyes was 6/9. Her left upper eyelid was mildly edematous with mild ptosis of 2 mm. Conjunctiva was injected. Otherwise, there was no anisocoria or relative afferent pupillary defect. The bilateral fundus was normal with no disc swelling, and extraocular movement was normal. The confrontation visual field test revealed a concentric contraction field defect over the left eye. Otherwise, the neurological examination was unremarkable. Secondary etiologies were excluded whereby her magnetic resonance imaging, magnetic resonance venography, and angiography brain were normal. She was referred to the neurology team and was diagnosed with hemicrania continua based on the criteria of the International Classification of Headache Disorders and resolution of headache with indomethacin.

**Figure 1:**
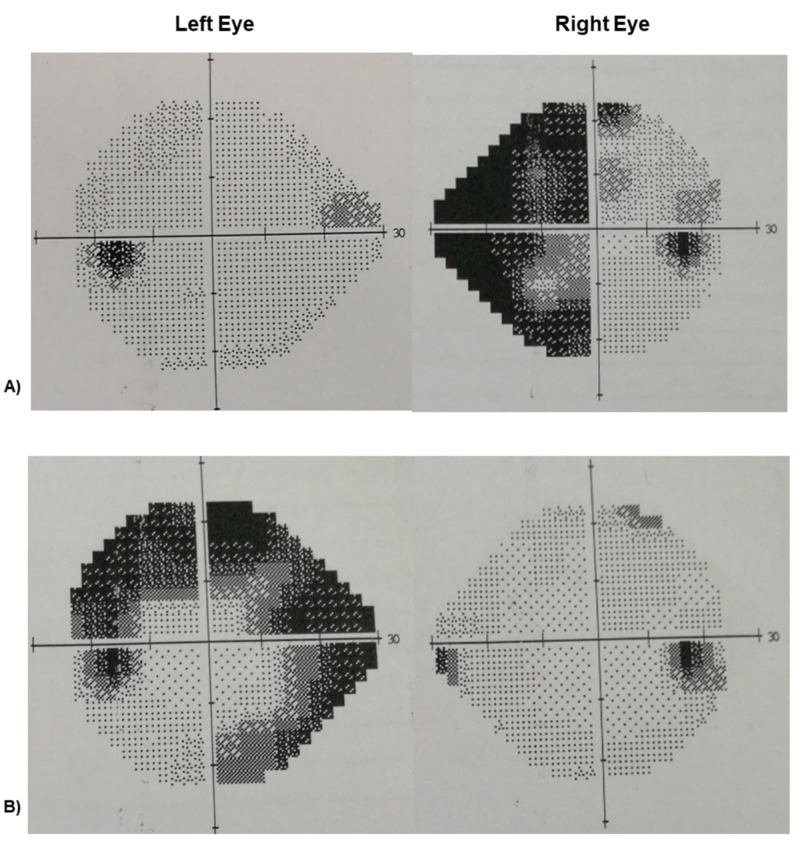
Sequential Humphrey 24-2 automated perimetric visual fields Performed at: A) One month during follow-up with right-sided headache exacerbation. There was a right nasal hemianopia. The left visual field was normal. B) 10 months. There was complete resolution of the right field defect and the presence of left ring scotoma during left-sided headache exacerbation.

Humphrey’s visual field test was done one month later during the follow-up visit where she developed a side-shifting headache affecting the right frontal and temporal region with features similar to the left side. The test revealed nasal hemianopia over the right eye and normal visual field over the left eye (Figure 1). A subsequent visual field test that was done 10 months later, while the patient was having a left-sided headache exacerbation, revealed left ring scotoma with complete resolution of the right eye field defect.

The patient was treated with indomethacin 50 mg bid initially and was gradually increased to 75 mg bid with a resolution of her headache, visual aura, and autonomic symptoms. Indomethacin could be tapered to 50 mg daily over the course of two months. Attempts to discontinue the medication resulted in a recurrence of pain and aura. She developed the side-effects of gastritis later and could not tolerate indomethacin. Her medications were changed to verapamil 40 mg tid, flunarizine 5 mg at night, and Sumatriptan 50 mg as needed. She had been followed up for the past 12 months. The intensity of the background headache was much improved (reduced from 4/10 to 2/10 on the subjective analog scale), with occasional weekly episodes of exacerbations as compared to the thrice-weekly exacerbations prior to treatment. The headache exacerbations predominantly affected the left side. Her visual aura remained the same during episodes of exacerbations.

## Discussion

Hemicrania continua (HC) is currently grouped under trigeminal autonomic cephalalgias (TACs) according to the International Classification of Headache Disorders (ICHD), based on the presence of prominent cranial autonomic features with worsening of headache, which seemed comparable to cluster headaches and paroxysmal hemicrania [1,4]. Based on the presence of the clinical phenotype of cranial autonomic symptoms and unilaterality, it was suggested that HC might share a pathophysiological similarity with TACs [5].

The mechanism for TACs is the role of trigeminal-autonomic reflex, which is a reflex pathway consisting of a brainstem connection between the trigeminal nerve and facial cranial nerve parasympathetic outflow [4,6-7]. A functional neuroimaging study of HC revealed the activation of the contralateral posterior hypothalamus and ipsilateral dorsal rostral pons in association with the headache [8]. A positive response to indomethacin is the typical feature of headache and diagnostic criteria peculiar to HC [1,4], as compared to other TACs.

Clinically, HC is characterized by a strictly persistent unilateral headache of moderate-intensity without a pain-free interval of more than three months, with episodes of exacerbations of severe intensity, which were associated with the presence of cranial autonomic features [2]. Apart from the presence of typical cranial autonomic features, which include conjunctival injection, lacrimation, nasal congestion, eyelid edema, and ptosis, as described by a clinical study done by Cittadini et al. [2], the most peculiar clinical finding in our patient was the complaint of visual aura in the form of transient scintillating visual scotoma that occurred during episodes of headache exacerbation, which was rare. To date, HC with associated visual aura has not been commonly reported. Peres et al. reported a case series of four patients with visual aura in the form of white flashes, sparkles, and black spots. The aura occurred preceding or during exacerbation of the headache, involving either the ipsilateral, contralateral, or both eyes [3]. From this case series, the author suggested that the aura might be pathophysiologically related to HC given the negative history of migraine in the patient and the family, as well as the response of both headache and aura to indomethacin. Recently, Auffenberg et al. described the first case of HC with classic scintillating scotoma lasting about 30 minutes during the headache exacerbation [9]. To our knowledge, our patient is the first reported case of HC with documented transient visual disturbance of scintillating scotoma ipsilateral to the side of headache exacerbation. Similar to the above case series reported, indomethacin gave complete relief of the headache, cranial autonomic features, and visual symptoms. In our patient, the visual aura and headache exacerbations were side-shifting, though affecting the left side most of the time.

Migrainous features were present in our patient but the diagnosis of migraine was less likely in view of the negative family history and the characteristic of unilateral headache associated with the presence of cranial autonomic features, which was more suggestive of a variant of TACs. The persistent nature of headache without a pain-free interval and dramatic response to indomethacin further favors the diagnosis of HC in this case. The visual symptoms described fulfilled the criteria of migraine with aura in ICHD. There were no visual symptoms observed independently of the headache. However, the resolution of both aura and headache with indomethacin suggests that there may be a pathophysiological correlation between aura and HC.

Table 1 lists the clinical features of trigeminal cephalalgias.

**Table 1:** Overview of clinical features of trigeminal cephalalgias

	Cluster headache	Paroxysmal hemicrania	Short-lasting unilateral neuralgiform headache attacks	Hemicrania continua
Sex (F:M)	1: 3	1: 1	1: 1.5	2: 1
Site of pain	Orbital, supraorbital, temporal	Orbital, supraorbital, temporal	Orbital, supraorbital, temporal, and/or other trigeminal distribution	Temporal, orbital, retro-orbital, frontal
Type of pain	Stabbing/throbbing	Stabbing/throbbing	Stabbing/throbbing	Baseline - dull pain. Exacerbations - throbbing, sharp during
Severity	Excruciating	Severe	Severe	Baseline – mild to moderate. Exacerbations - moderate to severe
Attack frequency	1 every other day – 8/day	>5/day	≥1/day	Continuous with exacerbations of moderate or greater intensity, or intermittent with remission period of ≥ 24 hours
Duration of attack	15 – 180 minutes	2 – 30 minutes	1 – 600 seconds	30 minutes to 3 days
Autonomic features	Yes	Yes	Yes	Yes
Migrainous features eg nausea, photophobia, phonophobia	++	++	+	+++
Indomethacin effect	No	Yes	No	Yes
Treatment	Oxygen, Sumatriptan	Indomethacin, Sumatriptan (partial relief)	No effective treatment	Indomethacin, Sumatriptan (partial relief)

## Conclusions

Strictly unilateral headache is a red flag. A detailed history and appropriate investigations are mandatory to exclude secondary causes. The diagnosis of HC is mainly clinical and remains a diagnosis of exclusion. It is a rare primary headache, which can present with side-shifting exacerbations, autonomic ocular features, and, rarely, a scintillating visual field defect as visual aura during episodes of exacerbation. Aura symptoms are not necessarily a specific feature of migraines. There may be a potential link with visual aura, which is currently not a diagnostic criterion of HC and needs to be further studied.
